# Molecular machines and devices

**DOI:** 10.3762/bjnano.7.29

**Published:** 2016-03-01

**Authors:** Jan van Ruitenbeek

**Affiliations:** 1 Huygens-Kamerlingh Onnes Laboratory, Leiden Institute of Physics, Leiden University, P.O. Box 9504, 2300 RA Leiden, The Netherlands

The suggestion to start this Thematic Series was first made at the symposium held on September 16–18, 2014 in Potsdam, Germany, under the same title, “Molecular Machines and Devices.” The format of this Beilstein Nanotechnology Symposium is intended to bring a relatively small group of specialists together for a few days to discuss progress and open challenges. The range of topics is chosen such that it is not too wide in order to have enough concrete overlap of interests and backgrounds, but at the same time, wide enough to bring researchers together from fields that are somewhat disjoint. The three main topics in the 2014 Potsdam symposium were: molecular electronics, one-dimensional conductors, and synthetic molecular machines.

Molecular electronics is currently being developed mostly at the interface between organic chemistry and nanophysics, leaning strongly towards the fundamental understanding of electron transport at the smallest scale and applications in nanoelectronics. The second topic, one-dimensional conductors, has been studied both theoretically and experimentally in the field of mesoscopic physics. More recently, truly one-dimensional wires, which are assembled from molecular building blocks into conductive oligomeric chains, have started to be investigated. This development provides a natural overlap between these two subfields of research. The third topic, synthetic molecular motors, has seen spectacular development in recent years, mostly in the research field of organic chemistry. In parallel, new theoretical ideas have been put forward by Tchavdar Todorov, Daniel Dundas, Mads Brandbyge, Per Hedegård, and others, which open the way for driving nanoscale motion by a dc current. The Potsdam meeting provided one of the first opportunities for researchers in each of these exciting but somewhat disconnected fields to interact and share ideas.

The format worked out beautifully. The number of participants, the setting at the shore of the beautiful Griebnitzsee, the diversity of research themes, the combination of theory and experiment, physics and chemistry, and the quality of the oral contributions all contributed to an atmosphere of enthusiasm and creativity. This can be judged from the fact that discussions continued over lunch and dinner and until late evening between participants with widely different backgrounds. One of the intriguing problems discussed included magnetic effects such as the giant, low field magnetoresistance observed at room temperature in organic nanowires presented by Wilfred van der Wiel [1]. The spin selectivity of transport through a helical organic wire presented by Ron Naaman [2] provided ample food for debate and led to new ideas for experiments and theoretical interpretation. There is also an interesting link between the theme of the symposium to recent observations in biology. Spiros Skourtis introduced the audience to the remarkable discovery of electron transport along micrometer long biomolecular wires, so-called pilli, growing as extracellular appendages from the surface of certain bacteria. The present understanding of the electron-conduction mechanism is described in terms of a hopping mechanism having remarkable properties [3]. These and many more topics are represented by the contributions to this Thematic Series.

This series presents the current research developments for many of these scientific problems and challenges. The works illustrate the interesting combination of theoretical questions, dedicated instrumentation development, synthetic chemistry, and experimental observations. Concepts for electrically actuated, molecular scale motion are discussed in many of the contributions, where switches, pumps, or motors are considered, and first experimental evidence is presented. The progress in theory and experiment on single-molecule electron transport can be judged from many contributions in this volume. On the other hand, some subjects are not as well-covered, including electron transport in biomolecules and one-dimensional conductors. This is merely the result of fortuitous fluctuations in having the proper material timely available for this volume, rather than a bias of interest.

Indeed, important developments can be anticipated at the interface with biomolecular sciences. In parallel, the first applications of molecular electronics are appearing on the market in the form of molecular layers. The top-contact problem, which has hindered applications for a long time, is now at least partially resolved, so that we may anticipate more applications to follow. These will initially be in the form of layers of single molecules. The path to single-molecule devices is still long, but there is a lot of interesting material to be discovered on the way.

Jan van Ruitenbeek

Leiden, February 2016
